# Supplementary material for: A pair of dopaminergic neurons DAN-c1 mediate Drosophila larval aversive olfactory learning through D2-like receptors
Source: eLife. 2025 Aug 13;13:RP100890. doi: 10.7554/eLife.100890 (PMC12349901; doi:10.7554/eLife.100890)
Supplement: Supplementary file 1. [file elife-100890-supp1.docx]

|  | **Strains** | **Information** | **Source/Gift** | **Stock #** |
| --- | --- | --- | --- | --- |
| **1** | R72C04 | D2R-GAL4 driver (verified) | BDSC | 49416 |
| **2** | R72C08 | D2R-GAL4 driver (verified) | BDSC | 49621 |
| **3** | R72D03 | D2R-GAL4 driver | BDSC | 46676 |
| **4** | D2R-EGFP | EGFP-tagged D2R | BDSC | 60276 |
| **5** | 201Y-GFP | GFP expressed in Mushroom Body | BDSC | 64296 |
| **6** | MB247-LexA::Vp16/TM6B | MB-LexA, mushroom body driver | Dr. M. Gallio's Lab | Macpherson et al. 2015 |
| **7** | UAS-nSyb-spGFP1-10,LexAop-CD4-spGFP11/CyO | GRASP strain | BDSC | 64314 |
| **8** | LexAop-nSyb-spGFP1-10,UAS-CD4-spGFP11;MKRS/TM6B | Reverse GRASP strain | BDSC | 64315 |
| **9** | WT(Canton-S) | Wild type | BDSC | 64349 (1) |
| **10** | UAS-RNAi(D2R) (III) line 2 | UAS-D2R-RNAi | Dr. A. Kopin's Lab | Draper et al. 2007 |
| **11** | 201Y-GAL4 | MB-GAL4, mushroom body driver | BDSC | 4440 |
| **12** | CyO/Sco;TM2,Ubx/TM6,Hu.Tb | Balancer chromosomes | Dr. S. Tanda's Lab |  |
| **13** | 1407-Gal4 | Pan-neuronal driver | BDSC | 8751 |
| **14** | 10XUAS-mCD8::GFP (I) | GFP strain | BDSC | 32189 |
| **15** | 10XUAS-mCD8::GFP (II) | GFP strain | BDSC | 32186 |
| **16** | 10XUAS-mCD8::GFP (III) | GFP strain | BDSC | 32184 |
| **17** | UAS-ChR2; UAS-ChrR2 | Optogenetics strain | Dr. B. Condron's Lab |  |
| **18** | LexAop-rCD2::RFP; UAS-CD4-spGFP1-10, LexAop-CD4-spGFP11 | RFP expressed in MB; GRASP | BDSC | 58755 |
| **19** | UAS-CD8::mCherry (II) | mCherry strain | BDSC | 27391 |
| **20** | UAS-CD8::mCherry (III) | mCherry strain | BDSC | 27392 |
| **21** | yw1118 | Wild type for D2R-EGFP | Dr. S. Tanda's Lab |  |
| **22** | D2R-miR | UAS-DD2R-microRNA | Dr. M. Wu's Lab | Xie et al. 2018 |
| **23** | UAS-*Shibirets1* | Thermogenetics strain | Dr. T. Kitamoto's Lab |  |
| **24** | UAS-dTRPA1 | Thermogenetics strain | BDSC | 26263 |
| **25** | UAS-nsyb::GFP; UAS-DenMark | Pre- and postsynaptic terminals | BDSC | 33056 |
