# Supplementary material for: A pair of dopaminergic neurons DAN-c1 mediate Drosophila larval aversive olfactory learning through D2-like receptors
Source: eLife. 2025 Aug 13;13:RP100890. doi: 10.7554/eLife.100890 (PMC12349901; doi:10.7554/eLife.100890)
Supplement: Supplementary file 2. [file elife-100890-supp2.docx]

| **Figure 1** | **N** | |  | **Figure S1** | **N** |
| --- | --- | --- | --- | --- | --- |
| **Strains** | **IF** | **GRASP** |  | TH-GAL4 | 4 |
| TH-GAL4 | 8 | 16 |  | MB065B | 4 |
| MB065B | 14 | 15 |  | R76F02AD;R55C10DBD | 5 |
| R76F02AD;R55C10DBD | 13 | 13 |  | MB296B | 3 |
| MB296B | 14 | 4 |  | SS1716 | 2 |
| SS1716 | 10 | 21 |  | R58E02 | 3 |
| R58E02 | 5 | 10 |  | R30G08 | 2 |
| R30G08 | 13 | 6 |  | SS1696 | 4 |
| SS1696 | 16 | 4 |  | SS0864 | 4 |
| SS0864 | 7 | 8 |  | SS1757 | 4 |
| SS1757 | 6 | 13 |  | R72D03-GAL4 | 7 |
|  |  |  |  | R72C08-GAL4 | 5 |
| **Figure 2** | **N** |  |  | R72C04-GAL4 | 3 |
| DAN-c1 x Syt-GFP;DenMark | 4 |  |  | 201Y-GAL4 | 4 |
|  |  |  |  | MB247-LexA::VP16 | 3 |
| **Figure 3** | **N** |  |  |  |  |
| DM1a | 11 |  |  | **Figure S2** | **N** |
| DM1b | 9 |  |  | TH-spGFP1-10 | 3 |
| pPAM | 11 |  |  | MB247-spGFP11 | 4 |
| DL1 | 10 |  |  |  |  |
| DL2a | 7 |  |  | **Figure S3** | **N** |
| DL2b | 5 |  |  | R72D03 | 5 |
| MB Soma | 6 |  |  | R72C08 | 4 |
| MB Lobe | 8 |  |  | R72C04 | 5 |
|  |  |  |  |  |  |
| **Figure 4** | **N** |  |  | **Figure S4** | **N** |
| DAN-c1 x D2-GFP | 9 |  |  | DAN-d1 x Syt-GFP;DenMark | 6 |
| TH-Gal4 | 7 |  |  | DAN-g1 x Syt-GFP;DenMark | 3 |
| TH-miR | 6 |  |  | DAN-d1 x D2-GFP | 13 |
|  |  |  |  | DAN-g1 x D2-GFP | 7 |
