# Supplementary material for: A pair of dopaminergic neurons DAN-c1 mediate Drosophila larval aversive olfactory learning through D2-like receptors
Source: eLife. 2025 Aug 13;13:RP100890. doi: 10.7554/eLife.100890 (PMC12349901; doi:10.7554/eLife.100890)
Supplement: Supplementary file 3. — For the strain R76F02AD; R55C10DBD, 22 third-instar larval brains expressing GFP or SytGFP and DenMark were examined, and all of them clearly identified DAN-c1. Half of them only identified DAN-c1, the rest had 1–5 weakly identified cells without neurites, and barely 1 or 2 strongly identified cells appeared. These non-DAN-c1 neurons were seldom dopaminergic neurons. In the ventral nerve cord (VNC), 8 out of 12 did not have any identified cells, 3 had 2–4 strong identified cells. These data supported that R76F02AD;R55C10DBD exclusively labels DAN-c1 in third-instar larval brains. [file elife-100890-supp3.docx]

| **R76F02AD;R55C10DBD (DAN-c1) brains** | **other DAN** | **None DAN** | **VNC** |
| --- | --- | --- | --- |
| **1** | 0 | 1 strong, 4 weak | NA |
| **2** | 1 weak no neurites | 0 | 3 weak |
| **3** | 0 | 7 weak | NA |
| **4** | 0 | 0 | 2 strong |
| **5** | 0 | 0 | 2 strong |
| **6** | 0 | 2 strong, 3 weak | NA |
| **7** | 0 | 0 | NA |
| **8** | 0 | 0 | 4 strong |
| **9** | 0 | 5 weak | NA |
| **10** | 0 | 1 weak | NA |
| **11** | 0 | 8 weak | NA |
| **12** | 0 | 0 | NA |
| **13** | 0 | 4 strong 1 weak | NA |
| **14** | 0 | 0 | 0 |
| **15** | 0 | 0 | 0 |
| **16** | 0 | 0 | NA |
| **17** | 0 | 0 | 0 |
| **18** | NA | 1 strong, 1 weak | 0 |
| **19** | NA | 7 weak | 0 |
| **20** | NA | 5 weak | 0 |
| **21** | NA | 0 | 0 |
| **22** | NA | 1 weak | 0 |
