# Supplementary material for: A pair of dopaminergic neurons DAN-c1 mediate Drosophila larval aversive olfactory learning through D2-like receptors
Source: eLife. 2025 Aug 13;13:RP100890. doi: 10.7554/eLife.100890 (PMC12349901; doi:10.7554/eLife.100890)
Supplement: Supplementary file 4. [file elife-100890-supp4.docx]

|  | **22°C** | | | **34°C** | | | **Interaction**  **p-value** | **Row Factor p-value (Temperature)** | **Column Factor p-value**  **(US)** |
| --- | --- | --- | --- | --- | --- | --- | --- | --- | --- |
|  | **QUI** | **DW** | **SUC** | **QUI** | **DW** | **SUC** |  |  |  |
| **shibire^ts1^** | 9 | 8 | 8 | 9 | 9 | 9 | 0.8880 | 0.5290 | <0.0001 |
| **DAN-c1×shibire^ts1^** | 14 | 6 | 7 | 17 | 6 | 9 | <0.0001 | - | - |
| **DAN-c1** | 8 | 9 | 9 | 9 | 9 | 9 | 0.6143 | 0.1664 | <0.0001 |
| **DAN-c1×dTRPA1** | 15 | 14 | 13 | 14 | 15 | 13 | <0.0001 | - | - |
| **dTRPA1** | 9 | 8 | 9 | 10 | 10 | 10 | 0.8001 | 0.4228 | <0.0001 |
