# Supplementary material for: A pair of dopaminergic neurons DAN-c1 mediate Drosophila larval aversive olfactory learning through D2-like receptors
Source: eLife. 2025 Aug 13;13:RP100890. doi: 10.7554/eLife.100890 (PMC12349901; doi:10.7554/eLife.100890)
Supplement: Supplementary file 5. [file elife-100890-supp5.docx]

| **Driver** | **Cross** | **Pentyl acetate**  **(PA)** | | | **Propionic acid**  **(P-A)** | | | **Naïve olfactory** | | **Naïve gustatory** | | **Locomotion** |
| --- | --- | --- | --- | --- | --- | --- | --- | --- | --- | --- | --- | --- |
|  |  | **QUI** | **DW** | **SUC** | **QUI** | **DW** | **SUC** | **PA** | **P-A** | **QUI** | **SUC** |  |
| **TH**  **×** | **miR** | 8 | 8 | 8 | 6 | 6 | 6 | 7 | 7 | 6 | 6 | 24 |
|  | **RNAi** | - | - | - | - | - | - | - | - | - | - | - |
|  | **CR** | 8 | 6 | 6 | 6 | 6 | 6 | 6 | 6 | 6 | 6 | 12 |
| **DAN-c1**  **×** | **miR** | 38 | 38 | 37 | 14 | 13 | 14 | 12 | 8 | 11 | 12 | 50 |
|  | **RNAi** | 6 | 6 | 6 | - | - | - | 8 | - | 6 | 6 | 24 |
|  | **CR** | 14 | 10 | 10 | 11 | 8 | 8 | 12 | 9 | 11 | 12 | 36 |
| **DAN-d1**  **×** | **miR** | 17 | 15 | 13 | 8 | 8 | 7 | 6 | 6 | 6 | 7 | 16 |
|  | **RNAi** | 6 | 6 | 6 | - | - | - | 7 | - | 7 | 6 | 16 |
|  | **CR** | 13 | 11 | 10 | 9 | 6 | 6 | 7 | 6 | 6 | 6 | 15 |
| **DAN-g1**  **×** | **miR** | 31 | 22 | 16 | 13 | 8 | 8 | 8 | 8 | 6 | 7 | 22 |
|  | **RNAi** | 8 | 8 | 6 | - | - | - | 8 | - | 6 | 7 | 24 |
|  | **CR** | 23 | 19 | 18 | 8 | 8 | 7 | 8 | 6 | 6 | 6 | 15 |
| **201Y**  **×** | **miR** | 7 | 7 | 8 | 6 | 6 | 6 | 6 | 6 | 6 | 6 | 24 |
|  | **RNAi** | - | - | - | - | - | - | - | - | - | - | - |
|  | **CR** | 6 | 6 | 6 | 6 | 6 | 6 | 6 | 6 | 6 | 6 | 24 |
| **WT** |  | 12 | 12 | 13 | 6 | 6 | 6 | 6 | 6 | 6 | 6 | 24 |
| **D2R-miR** |  | 12 | 12 | 12 | 6 | 6 | 6 | 12 | 6 | 11 | 12 | 36 |
| **RNAi** |  | 6 | 6 | 6 | - | - | - | 6 | - | 6 | 6 | 20 |
