# Supplementary material for: A pair of dopaminergic neurons DAN-c1 mediate Drosophila larval aversive olfactory learning through D2-like receptors
Source: eLife. 2025 Aug 13;13:RP100890. doi: 10.7554/eLife.100890 (PMC12349901; doi:10.7554/eLife.100890)
Supplement: Supplementary file 6. [file elife-100890-supp6.docx]

| **Strains** | **Groups** | **BL** | | | | | | | | | **No BL** | | |
| --- | --- | --- | --- | --- | --- | --- | --- | --- | --- | --- | --- | --- | --- |
|  |  | **Resting** | | | **Learning** | | | **Testing** | | |  |  |  |
|  |  | **QUI** | **DW** | **SUC** | **QUI** | **DW** | **SUC** | **QUI** | **DW** | **SUC** | **QUI** | **DW** | **SUC** |
| **TH×ChR2** | **ATR** | - | - | - | 21 | 13 | 10 | - | - | - | 20 | 16 | 10 |
|  | **No ATR** | - | - | - | 16 | 13 | 8 | - | - | - | 14 | 13 | 8 |
| **DAN-c1×ChR2** | **ATR** | 11 | 8 | 9 | 15 | 12 | 9 | 9 | 8 | 8 | 16 | 11 | 9 |
|  | **No ATR** | 9 | 9 | 8 | 15 | 12 | 8 | 9 | 9 | 9 | 16 | 12 | 9 |
| **201Y×ChR2** | **ATR** | 8 | 7 | 7 | 9 | 6 | 9 | 7 | 7 | 10 | 11 | 8 | 7 |
|  | **No ATR** | 10 | 7 | 7 | 10 | 7 | 7 | 9 | 7 | 13 | 12 | 10 | 7 |
